# Supplementary material for: Defining upper extremity dominance: The contributions of hand preference and grip strength
Source: PLoS One. 2025 Nov 25;20(11):e0336916. doi: 10.1371/journal.pone.0336916 (PMC12646442; doi:10.1371/journal.pone.0336916)
Supplement: Attachment — Submitted filename: response to the comment1.docx [file pone.0336916.s004.docx]

**Responses to the comments**

We very much appreciated your encouraging and insightful comments. We have endeavored to respond to all suggestions and comments, which further improved the understanding and potential impact of our paper. We responded to the mentioned comments in both the “revised manuscript” file and this one. In the manuscript, responses to the first reviewer have been highlighted green and yellow for the second reviewer. Hope our effort meets the editorial board's expectations.

Sincerely Yours,

Authors.

***Reviewer: 1***

**Authors:** Dear Professor, thank you very much for your time and valuable comments. I truly appreciate your helpful feedback. I have done my best to revise the text based on your suggestions and believe the changes have strengthened the work.

Have the authors made all data underlying the findings in their manuscript fully available? No

**Authors:** We confirm that all raw data underlying the findings of this study have been uploaded as a supplementary file in the journal’s submission system.

I perceive a restriction in the data availability from the statement of availability of data and materials. "The datasets utilized and/or analyzed during the current study are available from the corresponding

author upon reasonable request." The authors may need to clarify this.

**Authors:** We appreciate the reviewer’s observation. To clarify, all raw datasets generated and analyzed during the current study have been “uploaded as a supplementary file” in the journal’s submission system and are therefore “fully available”. The previous statement regarding data availability has been revised accordingly to eliminate any ambiguity.

Methods

The authors may have to specify if they used a sampling technique in this study. In this study, purposive sampling could be applicable and the reasons for its application stated clearly.

**Authors:** We thank the reviewer for this helpful comment. The study indeed employed a purposive sampling technique, as participants were deliberately selected based on specific inclusion criteria relevant to the study objectives. The “Participants” section has been revised to reflect this clarification.

Ethics approval and statement of consent to participate

"All participants signed a written informed consent form." For such a study, it would be a good practice leave a copy of the signed consent form with the study participant, and one retained by the researcher. The authors may also clarify this.

**Authors:** Dear Professor, it is don and highlighted in the manuscript.

***Reviewer: 2***

**Authors:** Dear Professor, thank you very much for your time and valuable comments. I truly appreciate your helpful feedback. I have done my best to revise the text based on your suggestions and believe the changes have strengthened the work.

This is More of the Background of the study, rather than Introduction. Add relevancy of the study and or problem statement. Ad a paragraph on Global and regional picture.

**Authors:** Dear Professor, it is don and highlighted in the manuscript.

AI generated. Even when you use AI to generate a text, try to read through and use human language.

**Authors:** Dear Professor, thank you for your consideration. This section has been revised and highlighted in the manuscript.

Enrich this section.

**Authors:** Dear Professor, we have revised this section and highlighted the changes in the manuscript.

Why 6 months? a trauma for last 8 months may also affect performance of my hand.

**Authors:** We thank you for this valuable comment. Following your suggestion, the exclusion period has been extended to one year to ensure full recovery and minimize any residual effects on hand function. It should be noted that only one participant reported a previous hand injury and was therefore excluded from the study. This revision has been incorporated and highlighted in the manuscript.

How did you select participants? How physically were they fit? What measure was used to test or ascertain physical fitness.

**Authors:** Great comment. It is added and highlighted in the manuscript.

Repetitive citation. Reference 11 is Carcia, yet you cited Garcia. Secondly, you can either choose to use one referencing style, Either Havard, or APA, or Vancouva. Just be consistent.

**Authors:** It is done.

All is AI-generated.

**Authors:** Dear Professor, it is don and highlighted in the manuscript.
