# Supplementary material for: Defining upper extremity dominance: The contributions of hand preference and grip strength
Source: PLoS One. 2025 Nov 25;20(11):e0336916. doi: 10.1371/journal.pone.0336916 (PMC12646442; doi:10.1371/journal.pone.0336916)
Supplement: Attachment — Submitted filename: response_to_the_comment1_auresp_2.docx [file pone.0336916.s005.docx]

**Responses to the comments**

Please state whether the data has been deposited in a public database/repository and indicate the data access license policies.

**Authors:**

We thank the reviewer for this important comment. The full dataset underlying the findings of this study has now been deposited in the Zenodo repository and is publicly available under a Creative Commons Attribution (CC BY 4.0) license. The data can be accessed at the following DOI: https://doi.org/10.5281/zenodo.17436917

The Data Availability Statement in the revised manuscript has been updated accordingly.
